# 

**Published:** 1880-11-20

**Authors:** 


### EDITORIALS AND MISCELLANEOUS.

*Cincinnati Colleges.*—There are reported no less than nine Medical Colleges in Cincinnati, of which only five are considered reputable.

*South-West Virginia Medical Society.*—A new medical organization has been organized in the South-west portion of Virginia. It will be auxiliary to the State Medical Society. The following gentlemen have been elected officers for the ensuing year:

*President*—W. F. Barn, M. D., L. L. D., Abingdon, Va.

*Vice-President*—1st, J. M. Estill, M. D., Tazewell C. H.; 2d, H. G. Johnston, M. D., Giles county; 3d, Oscar Wiley, M. D., Salem; 5th, J. L. Tipton, M. D., Hillsville; 5th, T. D. Kernan, M. D., Lebanon; 6th, J. T. Jordan, M. D., Newbern.

*Recording Secretary*—Geo. E. Wiley, M. D., Emory, Washington county.

### *OUR JOURNAL.*

As the year approaches its close, we want to say a few friendly words to our subscribers. The past year, though attended with marked prosperity in most lines of business, has been unusually hard upon Journals, by reason of the largely increased price of paper and material, while the rates of subscription remained the same. True, our list has increased, and the Journal has maintained its popularity with the profession; and yet we have to state that a large number of our subscribers are unmindful of our pressing necessities, and subject us to much needless embarrassment by simply neglecting to remit the small amount due from each one for subscription. We now kindly appeal to each and every one who is in arrears to remit us their subscription, and to renew for the coming year. The country is now everywhere prosperous, and certainly any physician can devote the proceeds of a single professional visit to procuring a Medical Journal for twelve long months. There is nothing so cheap, for the amount of usefulness and interest which it affords, as a Medical Journal to the practitioner of medicine, and it is a matter of astonishment that so many physicians are without a Medical Journal, and as a consequence are ignorant of the advances of the profession, and must practice at a disadvantage to themselves and their patrons, who have a right to expect the latest and most improved methods of treatment from those who assume the position and duties of a practitioner of medicine.

Judging from the notes of approval that frequently come up from our readers, our Journal is adapted to the wants of a large majority of the profession. We could give hundreds of these unsolicited testimonials, were it necessary, or proper, to do so, but submit only the following extract from one but recently received, which we do more on account of the sentiment it contains as to the duties of the profession than from any desire to make known the compliment conferred upon our Journal. The letter is from Dr. F. P. Akers, of DeKalb county, Ga., and we trust he will excuse the liberty we take in publishing it:

"I find your Journal exceedingly useful. It contains more practical matter in less space than any similar work with which I am acquainted,

The profession in the South ought to be proud of it, and should by all means sustain it. I don't see how any conscientious physician can do without a Medical Journal, and yet I know of practitioners who do not take a Journal, and who seldom or never read anything They make no progress, and the people know it. Such men may run for a time on gas and bluster, but they never wear well, and are sure to find, in a short time, their true level in public estimation.

